# Why do platelets express K^+^ channels?

**DOI:** 10.1080/09537104.2021.1904135

**Published:** 2021-04-19

**Authors:** Joy R Wright, Martyn P. Mahaut-Smith

**Affiliations:** 1 Department of Cardiovascular Sciences, University of Leicester, and NIHR Leicester Cardiovascular Biomedical Research Unit, Leicester, UK; 2 Department of Molecular and Cellular Biology, University of Leicester, Leicester, UK

**Keywords:** Platelets, potassium channel, membrane potential, megakaryocyte, intracellular Ca^2+^

## Abstract

Potassium ions have widespread roles in cellular homeostasis and activation as a consequence of their large outward concentration gradient across the surface membrane and ability to rapidly move through K^+^-selective ion channels. In platelets, the predominant K^+^ channels include the voltage-gated K^+^ channel Kv1.3, and the intermediate conductance Ca^2+^-activated K^+^ channel KCa3.1, also known as the Gardos channel. Inwardly rectifying potassium GIRK channels and KCa1.1 large conductance Ca^2+^-activated K^+^ channels have also been reported in the platelet, although they remain to be demonstrated using electrophysiological techniques. Whole-cell patch clamp and fluorescent indicator measurements in the platelet or their precursor cell reveal that Kv1.3 sets the resting membrane potential and KCa3.1 can further hyperpolarize the cell during activation, thereby controlling Ca^2+^ influx. Kv1.3^-/-^ mice exhibit an increased platelet count, which may result from an increased splenic megakaryocyte development and longer platelet lifespan. This review discusses the evidence in the literature that Kv1.3, KCa3.1. GIRK and KCa1.1 channels contribute to a number of platelet functional responses, particularly collagen-evoked adhesion, procoagulant activity and GPCR function. Putative roles for other K^+^ channels and known accessory proteins which to date have only been detected in transcriptomic or proteomic studies, are also discussed.

## Introduction

To date, approximately 20 functional ion channels have been clearly demonstrated in the platelet or its precursor cell, the megakaryocyte [1,2]. These include two potassium-selective ion channels, Kv1.3 and KCa3.1, which have been reasonably well characterized electrophysiologically (Table I) with proposals put forward for their biological function. In addition, a number of K^+^ channel-forming subunits or channel accessory proteins have been suggested to be present in the platelet through detection of mRNA transcripts, proteomics or antibody-based techniques (Table II). In this review, we summarize the evidence for various proteins that form or modulate K^+^-selective channels in the platelet and discuss their functional relevance. Excluded from this review are other proteins that contribute to membrane K^+^ permeability, such as nonselective ion channels (e.g. P2X1, ionotropic glutamate receptors, gap junction proteins) and transporters (e.g. Na^+^/K^+^ ATP-ase and the Na^+^/Ca^2+^/K^+^ exchanger).

**Table I.** Estimated channel densities per platelet: electrophysical and proteomic quantification

Potassium Channel Protein name	Channel Gene name	Level of detection	Patch clamp configuration	Maximum current or conductance (physiological saline)	Single channel current or conductance (extracellular [K^+^])	Estimated channels per platelet (electrophysiological quantification)	Estimated copies per platelet (Proteomic quantification) [3]	References
Voltage-gated potassium channel alpha subunit, Kv1.3	KCNA3	E P T	Plt whole cell	~100 pA (+40 mV)	0.35 pA (−40 mV) 9pS (5 mM K^+^)	≈285	<500	[2–8]
Intermediate conductance calcium-activated potassium channel protein 4, KCa3.1, Gardos channel, SK4	KCNN4	E P	Plt whole cell	Not measured	30pS (154 mM K^+^) 5pS (5 mM K^+^)	5–7	n.d.	[9–13]

E = electrophysiological investigation; *P* = proteomic or experimental study at protein level; T = transcriptomic level detection; n.d. = not determined

**Table II.** Additional potassium channel and channel regulatory proteins reported in human platelets

Potassium Channel-Associated Regulatory Protein name	Channel Gene name	Pore-forming protein or Regulatory protein	Level of detection	Estimated copies per platelet (Proteomic quantification) [3]	References
G-protein-activated inward rectifier potassium channel GIRK 1 (Kir3.1) GIRK 2 (Kir3.2) GIRK 4 (Kir3.4)	KCNJ3 KCNJ6 KCNJ5	Pore-forming Pore-forming Pore-forming	P P P	n.d. n.d. n.d.	[14] [14] [14]
Potassium channel subfamily K member 6, TWIK-2	KCNK6	Pore-forming	P T	840	[2,3,7,8,15]
Calcium-activated potassium channel subunit alpha-1, KCa1.1	KCNMA1	Pore-forming	P T	n.d.	[2,8,16]
Calcium-activated potassium channel Subunit beta-1 Subunit beta-2 Subunit beta-3	KCNMB1 KCNMB2 KCNMB3	Regulatory protein Regulatory protein Regulatory protein	T T T	n.d. n.d. n.d.	[2, 2, 2, 8]
Voltage-gated potassium channel Subunit beta-1 Subunit beta-2	KCNAB1 KCNAB2	Regulatory protein Regulatory protein	T P T	n.d. 1300	[2, 2, 3, 6]
Potassium voltage-gated channel subfamily E member 3, MiRP2	KCNE3	Regulatory protein	T	n.d.	[2,6,8]
Potassium channel regulatory protein	KCNRG	Regulatory protein	T	n.d.	[2,8]

*P* = proteomic or experimental study at protein level; T = transcriptomic level; n.d. = not determined

**A note on methodologies for assessing K^+^ channel activity in platelets including comparison with other myeloid cells**

Due to their fragile nature and small size, the number of direct patch clamp studies of the mammalian platelet remains limited (reviewed in [1]). Megakaryocytes are often used as a substitute for electrophysiological studies, and there is good evidence to suggest that the mature precursor cell is essentially a “giant” nucleated platelet [17,18]. Nevertheless, caution should be taken, particularly in terms of the detailed properties of channel activation, due to the substantial morphological rearrangements that take place during thrombopoiesis. While patch clamp is considered the “gold standard” when assessing ion channel properties, the challenge of applying this approach in the platelet means that much of the literature has assessed channel presence and contribution using less direct techniques. These include voltage-sensitive dyes, Rb^+^ flux measurements (since K^+^ channels are normally also permeable to Rb^+^, which can be measured using a radioactive isotope or a nonradioactive assay), proteomics and antibody-based approaches such as immunohistochemistry [3,4,9,14,19]. It is also worthwhile using a comparative approach as platelets and other blood cells are derived from a common stem cell within the marrow and studies of ion channels in other myeloid cells are substantially more advanced. While there are clear differences in channel complements between blood cell types (e.g. erythrocytes express KCa3.1 but not Kv1.3 [20,21]), there are major similarities, particularly regarding leukocyte K^+^ channels [20].

## KCa3.1

KCa3.1 (gene name *KCNN4*), also known as the Gardos channel and SK4, is a K^+^ selective ion channel activated by an increase in intracellular Ca^2+^. The channel is not activated by voltage at resting levels of Ca^2+^ and at elevated Ca^2+^ levels displays only minor increases in open probability in response to large depolarizations. It is often referred to as the intermediate conductance KCa channel due to the relative size of its single-channel conductance compared with other classes of Ca^2+^-activated K^+^ channel. Structurally, the channel consists of four identical subunits with each subunit comprising six transmembrane domains and a pore-forming domain [22]. Patch clamp experiments suggest that platelets express only a small number of functional KCa3.1 channels, around 5–7 per platelet [10], which may explain why the channel was not detected during transcriptomic screening of the platelet ion channelome [2]. The channel was first characterized in erythrocytes where it plays an important role in volume regulation [23].

The presence of Ca^2+^-dependent K^+^ channels in platelets was originally suggested from suspension measurements of membrane potential using the fluorescent indicator diSC_3_(5) [9,11,12]. The Ca^2+^ ionophore A23187 evoked a large hyperpolarization (a shift to a more negative membrane potential) that required external Ca^2+^ and was blocked by quinine, charybdotoxin (CTX), but not by apamin or tetraethylammonium [9], which are characteristics of KCa3.1 rather than small or large conductance Ca^2+^-gated K^+^ channels. There is some evidence for small conductance, apamin-sensitive KCa channels from Rb^+^ flux experiments [19]; however, these were not observed in whole-cell patch clamp recordings [10]. Direct electrophysiological studies in the platelet concluded that the channels are not active at resting levels of intracellular Ca^2+^ and reversibly stimulated by physiological increases in Ca^2+^, including repetitive transient Ca^2+^ spikes. Activation of this channel will therefore lead to membrane hyperpolarization toward the K^+^ equilibrium potential (~-90 mV) during agonist-evoked calcium signaling [10]. This agrees with the Ca^2+^-dependent activation characteristics of the channel in erythrocytes and leukocytes [24–26]. The threshold for stimulation by Ca^2+^ is approximately 200–300 nM, and maximal activation occurs at ~1 μM [Ca^2^]_i_. This dependence upon physiologically relevant levels of cytosolic Ca^2+^ has allowed the KCa currents to be used extensively in whole-cell patch recordings of megakaryocytes to investigate the mechanisms of Ca^2+^ oscillations [27].

Full platelet activation requires a sustained elevation of intracellular calcium, resulting in the externalization of the negatively charged membrane phospholipid component phosphatidylserine (PS) from the inner leaflet of the platelet membrane. Scott syndrome patients have a mild bleeding phenotype and have been noted to be deficient in the scramblase mechanism that facilitates PS exposure in erythrocytes and platelets, and also in the ability to produce platelet microparticles from the platelet surface membrane [28]. This defect in platelet procoagulant response may be due in part to reduced Gardos channel function, since the impaired procoagulant response in Scott patients following platelet activation with combined collagen and thrombin application was almost completely restored to normal levels by the K^+^ ionophore valinomycin [13]. Interestingly, valinomycin will insert into both intracellular and surface membranes [29] and could exert its observed effect at least in part by affecting mitochondrial membrane potential which has a key influence on procoagulant activity. Whether KCa3.1 is also present in platelet organellar membranes is unknown. Experiments in SK4^-/-^ transgenic mice suggest that the Gardos channel also plays a role in stromal cell-derived factor 1 (SDF-1)-dependent platelet migration [30]. A further potential role for KCa3.1 in platelets and megakaryocytes is the regulation of cell volume, as proposed in the human megakaryocytic cell line DAMI [31].

## Kv1.3

Kv1.3 (gene name *KCNA3*) is a voltage-gated K^+^ channel belonging to the Shaker-related subfamily. Structurally, it consists of four pore-forming homologous subunits, each consisting of six transmembrane alpha-helices, which also include the K^+^ selective pore and a voltage sensing domain [32]. Depolarization of the plasma membrane causes structural rearrangement of the voltage sensing domain, resulting in the opening of the conduction pathway.

Kv1.3 was originally reported in T lymphocytes, where the channel plays a role in mitogenesis [33]. Whole-cell patch clamp recordings have estimated between 200–300 Kv1.3 channels per cell. Since then, Kv1.3 has been detected in a wide range of electrically excitable and nonexcitable cell types [34–37]. Currents typical of this channel were first observed more than 30 years ago in platelets from a number of mammalian species [4,5] and later shown to be carried by Kv1.3 using a transcriptomic screen of all Kv α-subunits in human platelets and whole-cell patch clamp recordings of Kv1.3-deficient megakaryocytes [6]. This conductance has been characterized in megakaryocytes of several species and shown to be suppressed by activation of certain G-protein-coupled receptors, in part through a Gαi-coupled mechanism [38–41]. Each platelet clearly expresses considerably more Kv1.3 than KCa3.1 channels (almost 300 compared with 5–7 in humans) [4,5]. In physiological levels of K^+^, the single-channel conductance of Kv1.3 (~9pS for the predominant state) is slightly greater than for KCa3.1 (~5 pS). Thus, of these two main K^+^ conductances, Kv1.3 could potentially exert greater effects [10]. However, it is worth noting that the activation of KCa3.1 can hyperpolarize the membrane potential to levels that completely inactivate Kv1.3.

Kv1.3 has a threshold for activation of about −60 mV, thus accounting for its ability to set the resting membrane potential in both platelets and megakaryocytes [4,6]. Block of the channel with CTX or margatoxin causes a depolarization of ~25-35 mV from the resting membrane potential of −50 to −60 mV leading to a reduction in Ca^2+^ entry following stimulation of P2X1 receptors or store-operated Ca^2+^ channels [4,6]. This effect on Ca^2+^ influx can be explained by the depolarizing influence of Ca^2+^ or Na^+^ influx through agonist-activated cation channels and the fact that Kv1.3 is strongly activated by small depolarizations from the resting membrane potential [4,6].

Functional Kv1.3 channels have also been identified in the inner mitochondrial membrane (mitoKv1.3) [42,43]. MitoKv1.3 in lymphocytes has a role in maintaining mitochondrial membrane potential and regulates volume and reactive oxygen species production [42,44]. It has been suggested that mitoKv1.3 is a target for the pro-apoptotic protein Bax and is necessary for induction of apoptosis via the intrinsic pathway [43,45]. Platelets possess a small number of mitochondria and proteins belonging to the Bcl-2 family [46], but whether mitoKv1.3 is present in platelets and plays a role in platelet apoptosis has yet to be determined. However, there is good evidence for this hypothesis since Kv1.3^-/-^ mice have elevated levels of circulating platelets [6,47], and in our recent studies, a longer platelet lifespan was observed in Kv1.3^-/-^ mice [48]. Megakaryocyte development in the marrow was not increased [6,47], but a recent study has described enhanced megakaryocyte numbers in the spleen of Kv1.3^-/-^ mice [47], which may contribute to the enhanced platelet count.

In addition to its role as an ion channel, Kv1.3 may facilitate nonchannel functions since studies in lymphocytes have reported a direct interaction of Kv1.3 with β_1_ integrins, which regulates cellular adhesion [49–51]. Platelet adhesion and thrombus formation *in vitro* under conditions of arterial shear is significantly inhibited during perfusion of Kv1.3^-/-^ platelets over fibrillar collagen but not immobilized fibrinogen [47,48]. This specific role for Kv1.3 in α_2_β_1_-mediated adhesion is further supported by experiments using combinations of triple-helical collagen-specific peptides, whereby Kv1.3^-/-^ platelet adhesion is significantly reduced during perfusion over surfaces coated with von Willebrand factor (VWF)-III (a peptide that contains the VWF-A3 collagen-binding motif), and GFOGER (the α_2_β_1_-integrin-specific peptide), but not when perfused over surfaces coated with VWF-III and the glycoprotein VI-specific collagen peptide, CRP-XL [48]. Platelet exposure to collagen induces changes in platelet morphology, including the extension of filopodial protrusions to facilitate platelet attachment to collagen fibrils, followed by the formation of actin-rich lamellipodia and platelet spreading [52]. Kv1.3^-/-^ platelets display fewer filopodia per platelet than platelets from wild-type mice and additionally display a loss of directional persistence during chemotaxis toward the collagen fibrils [48], both of which may contribute to reduced numbers of adherent platelets and subsequently, the size of thrombus formed. Interestingly, Kv1.3 inhibition or deletion has previously been shown to alter the detection of electrical fields in neutrophils [53], and impair the migration of T-lymphocytes [54]. The ability of Kv1.3 to alter directional motility in platelets during α_2_β_1_-mediated adhesion may be through the interaction with β_1_ integrins (see above), possibly via mechanisms involving other regulatory proteins. Possible candidates for such regulatory proteins, including channel β-subunits or accessory proteins that bind to the cytosolic domain of Kv1.3, are discussed in more detail below. Further studies are required to fully understand the channel and nonchannel functions of platelet Kv1.3, and how Kv1.3 modulates platelet function in response to different agonists. Kv1.3 deletion has been reported to result in enhanced agonist-evoked platelet secretion in response to low-dose (1 and 10 µM) ADP, but has no effect on platelet secretion or aggregation in response to CRP-XL [48]. In contrast, genetic deletion or inhibition with the pore-blocking antibody (6E12#15) has been reported to reduce platelet aggregation in response to thrombin and collagen, and high-dose ADP (20 µM) [47]. Interestingly, both recent studies found no difference in *in vivo* thrombus formation or thrombus size in cremaster muscle arterioles of WT or Kv1.3^-/-^ mice following laser injury [48] or following FeCl_3_ injury in mesenteric arterioles [47]. Therefore, the lack of Kv1.3 may be compensated for *in vivo* by other channels or pathways; however, Kv1.3 channel inhibition with 5-(4-Phenoxybutoxy)psoralen (PAP-1) reduces infarct size and neurological scores in the middle cerebral artery occlusion model of ischemic stroke in rats [55]. The underlying basis of this inhibitory effect of PAP-1 is proposed to be through targeting Kv1.3 in microglia and a specific subset of central nervous system-infiltrating monocytes/macrophages involved in the inflammatory response of this model [55–57]. Since ischemic stroke is now recognized to be a thrombo-inflammatory disease [58], future studies should assess whether block of Kv1.3 in platelets also contributes to the potential therapeutic effect of targeting this channel.

## GIRK Channel

GIRK channels (Kir3.0 family) are G-protein-gated inwardly rectifying potassium channels. The rectification displayed by these channels results in an increase in conductance as the membrane is hyperpolarized. Thus, when activated, GIRK channels are very effective at shifting the membrane potential toward the equilibrium potential for K^+^ (≈-90 mV). Structurally, GIRK channels are tetrameric complexes consisting of 4 GIRK subunits 1–4, whereby each subunit comprises two transmembrane helices on either side of a pore-forming helix [59]. GIRK channel gating requires the presence of phosphatidylinositol 4,5 bisphosphate (PIP_2_), and interaction is sensitive to intracellular pH, sodium levels and arachidonic acid [60]. The channels are also modulated by G_α_ and G_βγ_ G-protein subunits, and GIRK channels have been reported to be present with GPCRs in macromolecular complexes [61].

One study has reported that platelets express GIRK1, GIRK2 and GIRK4 [14] and that two GIRK antagonists (SCH23390 and U50488H) inhibited platelet aggregation in response to ADP, meSADP, U46619 and low-dose thrombin, but not high-dose thrombin or convulxin. In contrast, the same GIRK inhibitors had no effect on G_q_ signaling-associated responses. Supporting this, studies in murine platelets that have defective GIRK2 function reported impaired ADP-induced TXA_2_ generation [62]. However, no impairment was observed in murine platelets where GIRK2 was absent, suggesting that the absent GIRK2 subunits were replaced by other GIRK subunits to maintain function. Further studies are needed to explore how these channels interact with each other, and with other proteins, to establish their possible role in the modulation of P2Y_12_ signaling in the platelet and validate functional channel activity via electrophysical recording.

## KCa1.1

KCa 1.1, encoded by the gene *KCNMA1*, is a large conductance calcium-activated K^+^ channel also known as BK, Maxi-K and Slo1. The functional channel is formed by a tetrameric assembly of alpha subunits, which can be associated with beta subunits that modify its function [63]. The opening of the channel is stimulated (gated) independently and synergistically by an increase in cytosolic Ca^2+^ and membrane depolarization. A very recent study has detected KCa1.1 using antibody-based techniques in human platelets and megakaryocytes [16]. Agonists (openers) of the channel exert an inhibitory effect on several functional responses, including aggregation or adhesion of platelets and proplatelet formation or cell spreading in megakaryocytes. The openers induce membrane hyperpolarization, as expected for an increase in relative permeability to K^+^. These results agree with an earlier study in which epoxyeicosatrienoic acids known to be released from endothelial cells induced a membrane hyperpolarization of human platelets that was blocked by iberiotoxin, a relatively selective KCa1.1 inhibitor [64]. Pharmacological openers of KCa1.1 also reduced the cytosolic Ca^2+^ responses to ADP [16]. At present, it is unclear why the membrane depolarization observed with the block of Kv1.3 [6] (see earlier section) and hyperpolarisation following KCa1.1 activation both lead to a reduced agonist-evoked Ca^2+^ response. The role of membrane potential *per se* in platelet and megakaryocyte function clearly merits further study. Electrophysiological measurements of KCa1.1 channels in platelets or megakaryocytes are also awaited and may require studies in human samples as patch clamp of Kv1.3-deficient megakaryocytes failed to detect other voltage-gated K^+^ conductances; thus, there may be a species difference [6].

## Other Potential Platelet K^+^ Channels

In addition to the K^+^ channels discussed above, a quantitative transcriptomic analysis of the human platelet ion channelome suggests that other K^+^ channels and K^+^ channel regulatory proteins are expressed and thus may contribute to platelet function [2]. RNA transcripts for *KCNK6*, a 2-pore channel (other names TWIK-2, potassium channel subfamily K member 6), were detected at 7-fold lower level than Kv1.3 (*KCNA3*), and have also been reported at the protein level [3,7]. *KCNK6* is widely expressed in other cells and tissues and has been reported to contribute to vascular contractility [65]; it has also been identified as one of the triggers for macrophage NLRP3 (Nucleotide-binding oligomerization domain-Like Receptor containing Pyrin domain 3) activation of the inflammasome [66] and located in Lamp-1-positive lysosomes in transfected Madin-Darby canine kidney epithelial cells [67]. The identity of ion channels that reside in platelet lysosome membranes or play a role in platelet lysosomal secretion is poorly understood, and our understanding of molecular mechanisms involved in the platelet inflammatory response is still limited. Further validation and characterization of *KCNK6* may enhance our knowledge of platelet activation and responses. Platelet mRNA was also detected for *KCNMA1* (KCa1.1), the pore-forming subunit of the large conductance calcium-activated K^+^ channel and three of its regulatory subunits (*KCNMB1*,2 and 3). Over-expression of *KCNMA1* in human hepatic stellate cells (HSC) resulted in reduced migration, and Rotterlin activation of *KCNMA1* channels resulted in the downregulation of TGFB1/SMAD3 and JAK/STAT3 signaling pathways [68]. Meanwhile, a mutation in *KCNMA1* that reduced channel conductance and ion selectivity resulted in impaired mitochondrial function [69].

Transcripts were also detected in platelets for several β-subunits that modulate the pore-forming α-subunits of voltage-gated potassium channels, including the voltage-gated Shaker-related subunits (*KCNAB1,2*, and *3*). *KCNAB1* has been reported to modulate the channel activity of voltage-gated potassium α-subunits, promoting its expression at the cell membrane, but also accelerating the channel pore closure [70,71], possibly through the binding of NADPH [72]. *KCNE3* (MiRP2) from the Isk-related family is another β-subunit that has been reported to form complexes with the α-subunits of voltage-gated potassium channels, resulting in reduced current density and modulation of channel activation rates [73]. Another KCNE subunit (*KCNE4*, MiRP3) has been reported to retain Kv1.3 in the endoplasmic reticulum of leukocytes when the surface targeting motif of Kv1.3 COOH terminus is masked [74], and *KCNRG* (Potassium Channel Regulatory Protein) encodes a soluble protein that has been suggested interferes with the assembly of Kv channels, suppressing K^+^ currents [75,76]. Therefore, several candidates exist for β-subunits with regulatory roles in platelet K^+^ channel function that are worthwhile exploring in future studies.

Several members of the K^+^ channel tetramerization domain-containing proteins (KCTDs) family were also detected in the transcriptomic channelome study [2]. The biological roles of this large family of proteins are still being determined; however, in terms of ion channel-related roles, they have been reported to interact with GABAB GPCRs resulting in modulation of receptor sensitization [77]. The KCTD transcripts detected fell predominantly into the Group A and B subfamilies. To date, *KCTD10*, the most abundant KCTD transcripts quantified in the platelet, has been reported to bind to the T-box transcription factor Tbx5, a gene involved in cardiac development, and repress its transcriptional activity [78]. Furthermore, a single nucleotide polymorphism of *KCTD10* has been associated with variation of high-density lipoprotein cholesterol concentrations in individuals with high carbohydrate intakes [79]. Several KCTD family members have been reported to interact with cullin-3, leading to the degradation of specific target proteins [80], alteration of actin organization [81] and Rac1 activation [82]. Whether any KCTDs are present at the protein level and actively contribute to platelet function, in particular to K^+^ ion channel function, remains to be determined.

## Platelet- or Megakaryocyte-related K^+^ Channelopathies

The consequences of loss of Kv1.3 function based upon the use of blockers and Kv1.3^-/-^ mice are discussed above. Within such studies, it is worth considering that KCa3.1 and Cl^–^ channels are known to compensate for the lack of Kv1.3 in lymphocyte development and function [40]. One study has reported a loss of Kv1.3 channel function in megakaryocytes from a proportion of patients with myelogenous leukemia and reappearance of the channel after treatment [41]. Whether this shift in channel expression is a causal factor in the development of the leukemic phenotype is unknown. Interestingly, a number of human myeloid cell lines with megakaryocytic surface markers that have been derived from cancer patients also lack Kv1.3 but express robust KCa3.1 currents [2,83–85]. This represents a shift in K^+^ channel phenotype toward that of human erythrocytes rather than leukocytes or platelets [20,21]; thus, it is interesting to speculate that a reprogramming toward a more erythroid-type lineage occurs within megakaryoblastic leukemias. Indeed, observations of changes in conductance with megakaryocyte differentiation have led to the suggestion that in patients with myelogenous leukemias, these cells display a more immature or dedifferentiated form [41]. Under resting conditions, the loss of Kv1.3 but retention of KCa3.1 will yield a reduction in homeostatic K^+^ fluxes and a depolarization of the membrane potential of ~30 mV; however, the ability to hyperpolarize to near the K^+^ equilibrium potential of ≈-90 mV following an elevation of cytosolic Ca^2+^ will be retained [4,6,85].

Alterations to platelet Ca^2+^-dependent K^+^ channel function have been suggested to occur in Alzheimer’s disease [86]. This neurodegenerative disease is characterized pathologically by the appearance of proteinaceous plaques in areas of the brain [87]. Indeed, platelets represent a principal peripheral source of a major plaque component, β-amyloid, and its precursor, β-amyloid precursor protein [88,89]. The relative role of platelet sources of these proteins during Alzheimer’s is unclear; however, the properties of platelet K^+^ channels could be markers for early stages of this debilitating disease, which is becoming a significant increasing problem in the aging community of the Western world.

## Summary and Conclusion

**Figure 1.**
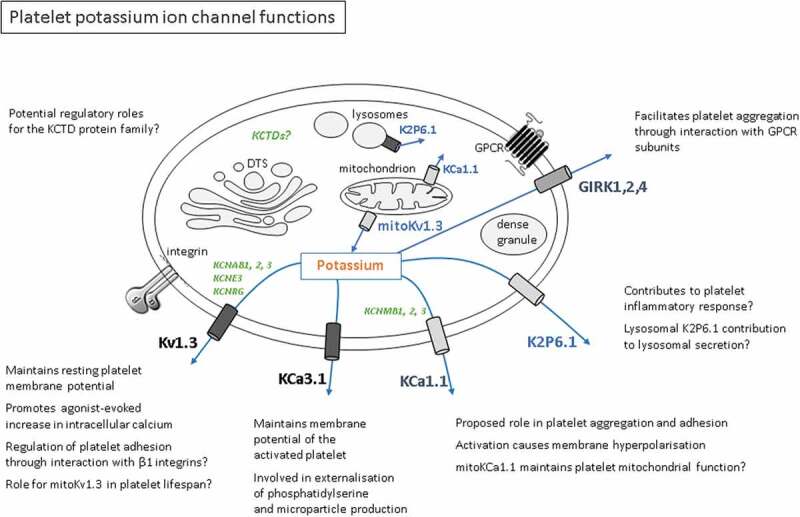
Platelet potassium ion channel functions

While there is currently a paucity of information on the contribution of K^+^ channels to platelet function, it is clear that they control the membrane potential under resting and activated conditions. This will have a major influence on Ca^2+^ influx, particularly given the inwardly rectifying nature of store-operated Ca^2+^ channels [90,91], and thus, in theory modulate the wide range of Ca^2+^-dependent responses in platelets including shape change, secretion, procoagulant activity and integrin inside-out signaling [92–95]. Roles in mitochondrial activity have been implied but not yet been clearly demonstrated [6]. More direct effects on procoagulant activity have also been reported. Given the major role of Kv1.3 and KCa3.1 in erythrocyte and leukocyte function, it is likely that additional roles for K^+^ channels in the platelet and megakaryocyte exist. In addition to an influence through control of membrane potential, these channels may directly interact with integrins and mitochondrial Bcl-2 proteins. The location and possible interactions of K^+^ channels with other proteins in the platelet are summarized in Figure 1. A further signaling mechanism that has been reported in leukocytes is via control of intracellular K^+^ and serine/threonine phosphatase activity, which can have a substantial effect on T cell development and function [95,96]. Given the established roles and therapeutic potential of K^+^ channels in other blood cells (e.g. treatment of autoimmunity and sickle cell anemia) [51,97], increasing our knowledge of this major class of membrane protein function and our understanding of their regulation in the platelet and its precursor cell could yield useful additional targets for modulation of thrombosis and other platelet-dependent diseases.

The diagram shows potassium channels that have been characterized by electrophysiological studies and biological function (black bold text: Kv1.3, and KCa3.1), or by platelet function only (gray bold text: GIRK1, 2, and 3 and KCa1.1); and potassium channels that have been identified by transcriptomic and/or proteomic studies of platelets but have not yet been further characterized (bold blue text: K2P6.1). Also shown (in green text) are gene names for platelet mRNA transcripts encoding regulatory β-subunits, which may modulate α-subunit channel function (*KCNAB1*, 2, and 3; *KCNE3; KCNRG; KCNMB1, 2*, and *3*; and the *KCTD* family). Text boxes in the diagram list known and potential roles for each ion channel.
